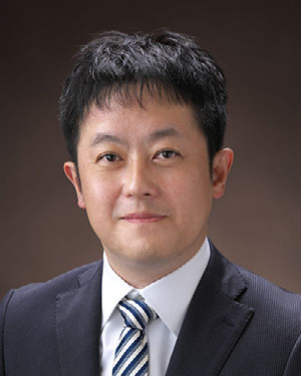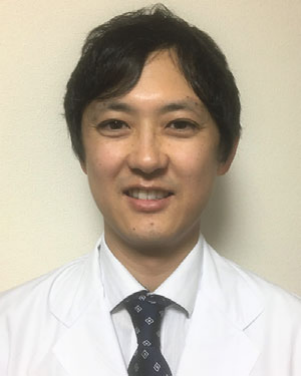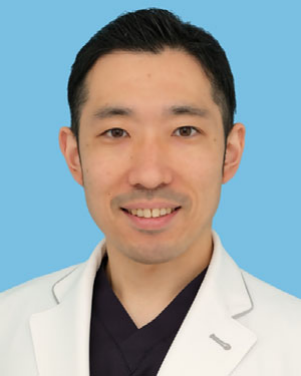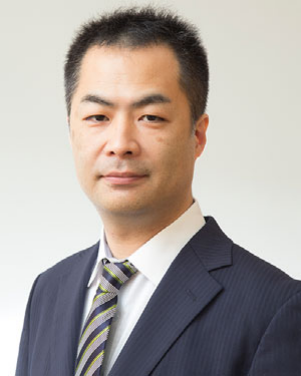# Best Reviewers Award for 2024

**DOI:** 10.1002/deo2.70101

**Published:** 2025-03-30

The DEN Open Best Reviewers Award is an annual prize that recognizes the very best reviewers for their high‐quality reviews and dedication. Over 271 scholars served as reviewers in 2024, and we are pleased to announce 20 winners who have been selected based on the following criteria:

1. Invitation acceptance rate: 80% and over;
2. The number of completed reviews calculated based on;
  - Review/Original Article/Techniques and Innovation ——— x 1
  - Case Report ——— x 0.5
3. Review quality scored for each review based on; 5, Excellent/4, Good/3, Average/2, Below average/1, Poor.
4. Top 20 or more reviewers to be awarded based on the criteria below;

Total number of reviews (for 2024: 1.5 or above).

Total score of review quality.

Review period: from 1 January 2024 to 31 December 2024

**Table:** 

**Akira Dobashi** Department of Endoscopy, The Jikei University Kashiwa Hospital, Chiba, Japan	**Osamu Dohi** Molecular Gastroenterology and Hepatology, Graduate School of Medical Science, Kyoto Prefectural University of Medicine, Kyoto, Japan	**Mitsuru Esaki** Division of Gastroenterology and Hepatology, Mayo Clinic Arizona, Scottsdale, Arizona, USA	**Kingo Hirasawa** Division of Endoscopy, Yokohama City University Medical Center, Kanagawa, Japan
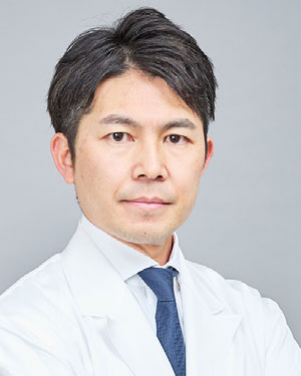	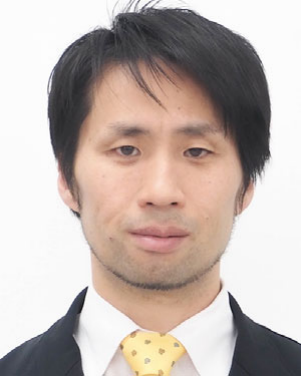	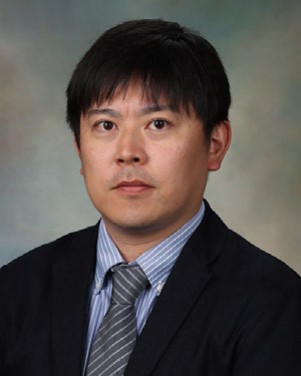	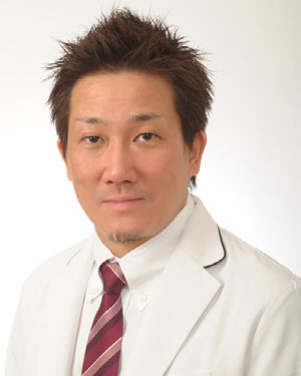

**Table:** 

**Takashi Hirose** Department of Endoscopy, Nagoya University Hospital, Aichi, Japan	**Yusuke Ishida** Department of Gastroenterology and Medicine, Fukuoka University Faculty of Medicine, Fukuoka, Japan	**Tomohiro Kadota** Department of Gastroenterology and Endoscopy, National Cancer Center Hospital East, Chiba, Japan	**Tsunetaka Kato** Department of Endoscopy, Fukushima Medical University Hospital, Fukushima, Japan
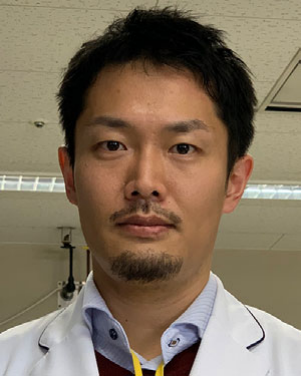	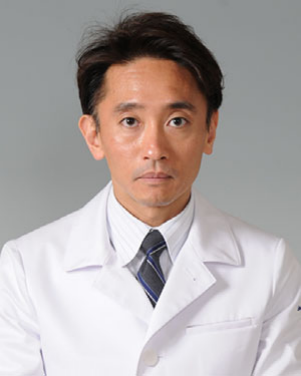	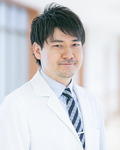	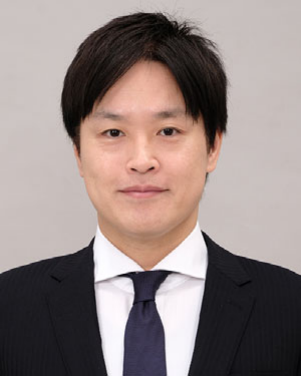
**Noriko Matsuura** Division of Research and Development for Minimally Invasive Treatment, Cancer Center, Keio University School of Medicine, Tokyo, Japan	**Kazunari Nakahara** Department of Gastroenterology, St. Marianna University School of Medicine, Kanagawa, Japan	**Jun Nakamura** Department of Endoscopy, Fukushima Medical University Hospital, Fukushima, Japan	**Yohei Ogata** Division of Gastroenterology, Tohoku University Graduate School of Medicine, Miyagi, Japan
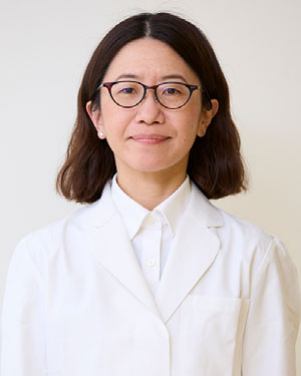	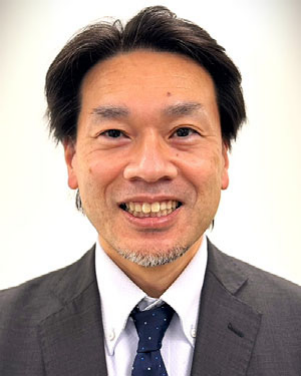	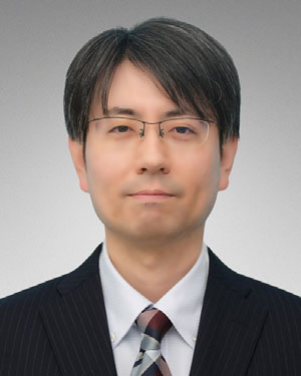	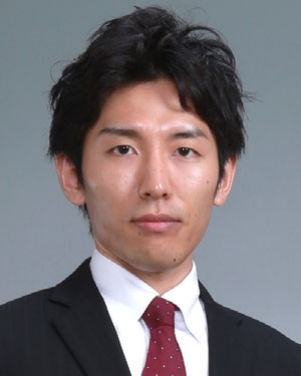
**Shunsuke Omoto** Departments of Gastroenterology and Hepatology, Kindai University Faculty of Medicine, Osaka, Japan	**Fumisato Sasaki** Digestive and Lifestyle Diseases, Kagoshima University Graduate School of Medical and Dental Sciences, Kagoshima, Japan	**Takeshi Tomoda** Department of Gastroenterology, Okayama City Hospital, Okayama, Japan	**Yosuke Toya** Division of Gastroenterology and Hepatology, Department of Internal Medicine, Iwate Medical University, Iwate, Japan
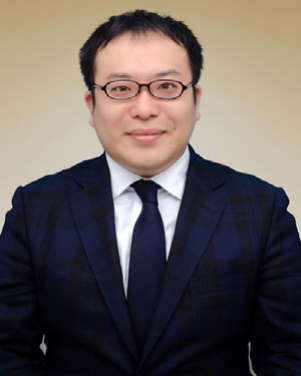	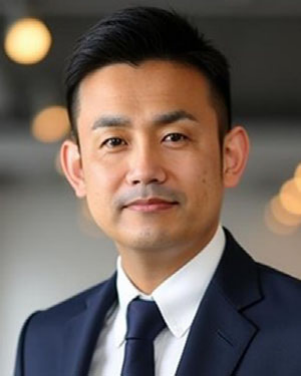	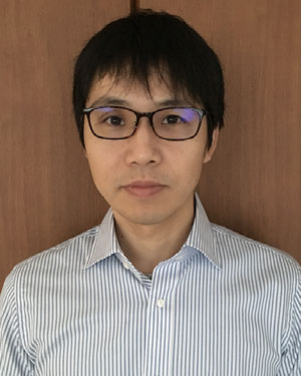	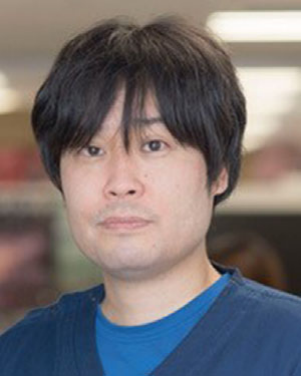

**Table:** 

**Daisuke Yamaguchi** Division of Gastroenterology, Department of Internal Medicine, Faculty of Medicine, Saga University, Saga, Japan	**Yasushi Yamasaki** Department of Gastroenterology, Okayama University Hospital, Okayama, Japan	**Takeshi Yasuda** Department of Gastroenterology, Akashi City Hospital, Hyogo, Japan	**Shinji Yoshii** Department of Gastroenterology and Hepatology, Sapporo Medical University School of Medicine, Hokkaido, Japan